# A computational-experimental investigation on high ethylene selectivity in ethanol dehydration reaction found on WO_*x*_/ZrO_2_-activated carbon bi-support systems

**DOI:** 10.1038/s41598-019-56373-3

**Published:** 2019-12-24

**Authors:** Meena Rittiruam, Bunjerd Jongsomjit, Supareak Praserthdam

**Affiliations:** 1 High-Performance Computing Unit (CECC-HCU), Center of Excellence in Catalysis and Catalytic Reaction Engineering (CECC), Chulalongkorn University, Bangkok, 10330 Thailand; 2 Center of Excellence in Catalysis and Catalytic Reaction Engineering (CECC), Chulalongkorn University, Bangkok, 10330 Thailand

**Keywords:** Heterogeneous catalysis, Chemical engineering

## Abstract

The high ethylene selectivity exhibited on the zirconia-activated-carbon bi-support catalyst is investigated by experiment and density functional theory–based (DFT) analysis. This bi-support catalyst systems prepared by the physical mixing method for the tungsten catalyst show a significant increase in ethylene selectivity up to 90% compared to the zirconia single support system (~58%) during the ethanol dehydration reaction. Besides, the optimal percent weight ratio of zirconia to activated carbon, which results in the highest ethanol conversion is 50:50. The DFT–based analysis is used to investigate high ethylene selectivity in the bi-support system. It shows that the WO_5_/zirconia is the most stable model for the zirconia single-support tungsten catalyst represented by the zirconia (101) facet of the tetrahedral phase. The carbon atoms were added to the WO_5_/zirconia to model the tungsten catalyst on the bi-support system. The Bader charge analysis is carried out to determine the electron transfer in the catalyst. The bonding between ethylene and the WO_5_ active site on the catalyst is weakened when the system is bi-support, where the added carbon atoms on the catalyst in the ZrO_2_ region decrease the ethylene adsorption energy. Thus, the desorption and the selectivity of ethylene are promoted. The decrease in adsorption energy can be explained via the analysis of the projected density of states (PDOS) profiles of atom involving the adsorption. It was found that the added carbon in the ZrO_2_ region induces the electron transfer from the ethylene molecule to the surface, especially to the ZrO_2_ region. The depletion of the electron around the ethylene molecule weakens the bonds, thus, promote desorption. Hence, the advantages of using the bi-support system in the tungsten catalyst are that the catalyst exhibit (1) high conversion due to the zirconia support and (2) high ethylene selectivity due to the added carbon promoting the desorption of ethylene via the induction of electron from an ethylene molecule to surface.

## Introduction

Ethylene, one of the essential products of ethanol dehydration, plays a critical role in the petrochemical industry with its total demand of more than 150 million tons per year^1,2^. Hence, the development of the ethylene industry is very important. Also, in many countries, the oversupply of ethanol is faced due to the policy promoting the use of electric vehicles, which in turn lowers the ethanol demand for the use as an octane-enhancing additive in gasoline. Therefore, the utilization of ethanol is of important. One process is to convert ethanol via a catalytic process of ethanol dehydration. The reaction is advantageous due not only to that it effectively produces ethylene but also contributes to the shifting of the typical fossil fuel-based process to the bio-based substitution^3^. Nowadays, several works have been studying the mechanism of the ethanol conversion reaction to produce ethylene together with their deactivation scheme on various catalysts, e.g., Al_2_O_3_^3–5^, MFI zeolites^6^, HZSM-5^7–9^, Ce_2_O_3_^10^. For the Al_2_O_3_ catalyst, although high selectivity and activity were found, the reaction temperature is high at about 380 °C^2^. Thus, the process is not power saving. In addition, the HZSM-5 suffered from the deactivation via coking caused by the ethylene polymerization reaction, which is the rate-limiting step reducing the rate of the main reaction^9^. For the Ce_2_O_3_ catalyst, the computational study revealed that acetaldehyde is the main product while ethylene is the main product at high temperature due to the changes in adsorbate and surface structure^10^. Besides, the experimental and theoretical study found that the diethyl ether (DEE) formation is the predominant pathway over the internal acid sites of the MFI zeolites, while, to produce ethylene, it still needs high temperature^6^. In addition, the zirconia shows high acidity and activity during the acid-catalyzed reaction, e.g., esterification reaction with high stability when it is used as the support in the tungsten oxide catalyst^11^. Also, the activated carbon known for its high surface area to weight ratio^12^ is the promising support to be used to enhance the distribution of the active site promoting the catalyst activity.

Hence, it is a good candidate to be used as the second component in the bi-support system. Moreover, there are a few related experimental works on zirconia and carbon discussed for state-of-the-art in those surface properties^13–16^. Therefore, we proposed to develop a high activity and selectivity solid acid catalyst to be used in the production of ethylene via the ethanol dehydration reaction utilizing the bi-support system comprising the nanocrystal zirconia and high surface area activated carbon.

The descriptors for the performance of the catalyst in this reaction are (i) the activity in terms of ethanol conversion and (ii) the product selectivity towards ethylene. Ultimately, we employed the density functional theory (DFT) to investigate the role of the activated carbon in the ZrO_2_ support for the tungsten catalyst exhibiting a high ethylene selectivity to understand and propose the guideline for the design of a more active and highly selective towards ethylene production.

## Results and Discussion

### Effect of activated carbon single support of tungsten catalyst

**Figure 1:**
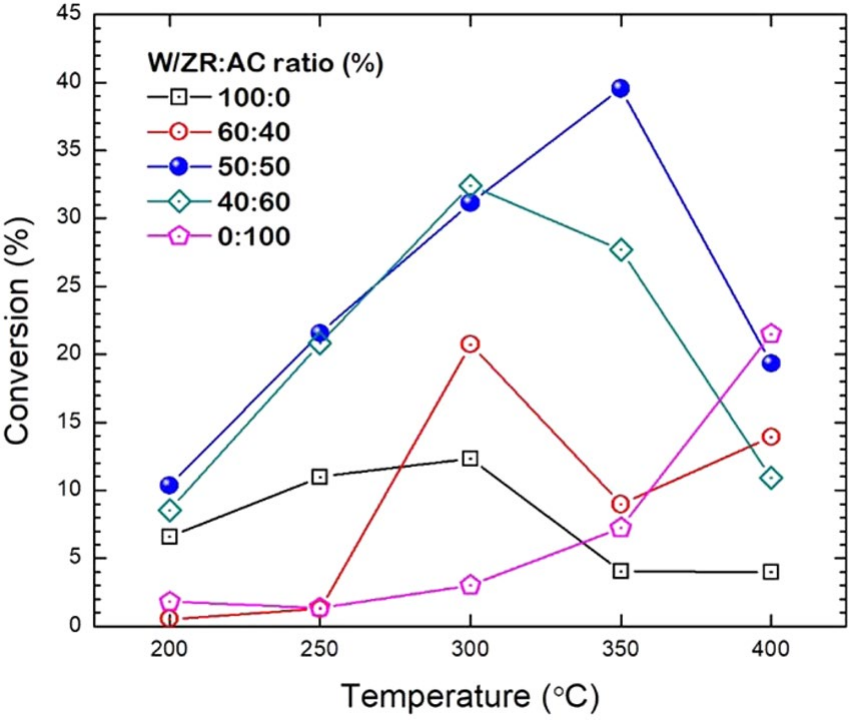
Conversion of WO_*x*_/ZrO_2_–AC catalysts at various temperatures.

In the first part, we compare the performance between the activated carbon and zirconia single-support systems. In Table 1, the activated carbon single support shows high surface area to weight, but still lower than the activated carbon synthesized to be catalyst^17^. Besides, its acidity is higher than that of the zirconia, which is considered advantageous to the activity of the acid-catalyzed reactions^18^. In Fig. 1, the comparison of single support catalyst showed that the W/ZR yielded the conversion more than W/AC from 200–300 °C, while, the W/AC shows high conversion than W/ZR at 450 °C. The 60:40 and 40:60 of W/ZR:AC exhibit the maximum conversion at 300 °C for around 21% and 33%, respectively. We found that the highest conversion is obtained by the 50:50 W/ZR:AC catalyst yielding at 350 °C.

**Table 1:** Characterizations of tungsten catalyst on various support systems.

Catalyst	Activated carbon content (wt %)	Surface Area (m^2^/g)	I_970_/I_805_	Acidity (μmol H^+^/g)
WO_*x*_/ZrO_2_	0	121	4.32	178
WO_*x*_/ZrO_2_60–AC40	40	91	2.67	75
WO_*x*_/ZrO_2_50–AC50	50	84	0.25	72
WO_*x*_/ZrO_2_40–AC60	60	67	1.39	140
WO_*x*_/AC	100	163	—	208

**Figure 2:**
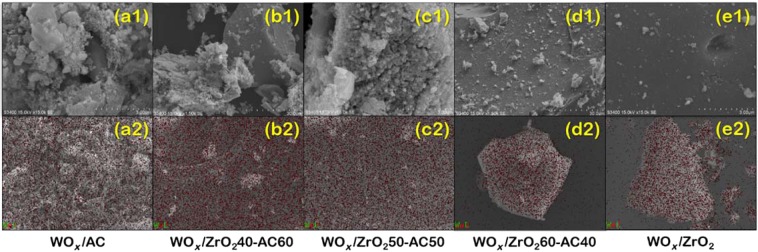
Morphology (a1, b1, c1, d1, e1) and EDX elemental mapping of WO_*x*_ catalyst on (**a**) activated carbon single support (WO_*x*_/AC), (**b**) zirconia 40% -activated carbon 60% bi-support (WO_*x*_/ZrO_2_40–AC60), (**c**) zirconia 50% -activated carbon 50% bi-support (WO_*x*_/ZrO_2_50–AC50) (**d**) zirconia 60% - activated carbon 40% bi-support (WO_*x*_/ZrO_2_60–AC40), and (**e**) zirconia single support (WO_*x*_/ZrO_2_). The EDX profile, projected onto each image for WO_*x*_, red point, is shown in a2–d2.

To describe the difference in the conversion of the single-support systems, where we hypothesized this to be from the difference in the deposition of the tungsten active sites on each surface, the SEM-EDX technique is used to investigate the surface morphologies and the distribution of the W on our catalyst. In Fig. 2(a1), we found that the WO_*x*_ is bigger than the pore of the activated carbon causing the pore blockage. Meanwhile, Fig. 2(b1) shows that the WO_*x*_ is highly distributed on the zirconia than on the activated carbon support. For this reason, the pore blockage caused by the WO_*x*_ in the activated carbon system resulting in low activity. Thus, its low conversion. Besides, the operating temperature of the ethanol dehydration reaction in the activated carbon system increased with temperature, while in the zirconia system, the conversion is also enhanced with temperature but higher than that of the activated carbon system significantly decreased above 300 °C.

Note that the coking occurred on the catalyst, which can be observed after the reaction, where the color of the zirconia changed from white to black. This also explains the decrease in conversion at the temperature above 300 °C. This shows that when using the zirconia as support, although the reaction is promoted more, the life of the zirconia is shorter than that of the activated carbon support.

### Effects of the bi-support system on the performance of the tungsten catalyst

In this part, the bi-support system comprises zirconia, and activated carbon prepared by the physical mixing method is studied for its effect on the tungsten catalyst comparing to the single-support system of both zirconia or activated carbon alone.

**Figure 3:**
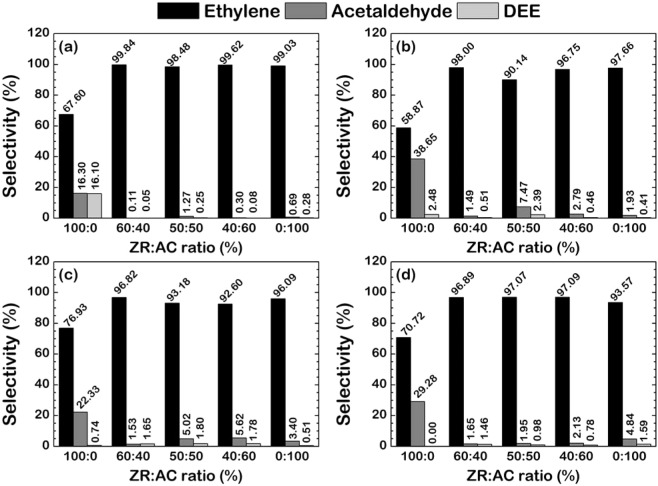
Products selectivity of WO_*x*_/ZrO_2_–AC catalysts at 250 °C (**a**), 300 °C (**b**), 350 °C (**c**), and 400 °C (**d**), where (1) Ethylene, (2) Acetaldehyde, and (3) Diethyl ether (DEE) are considered as dominant products.

**Figure 4:**
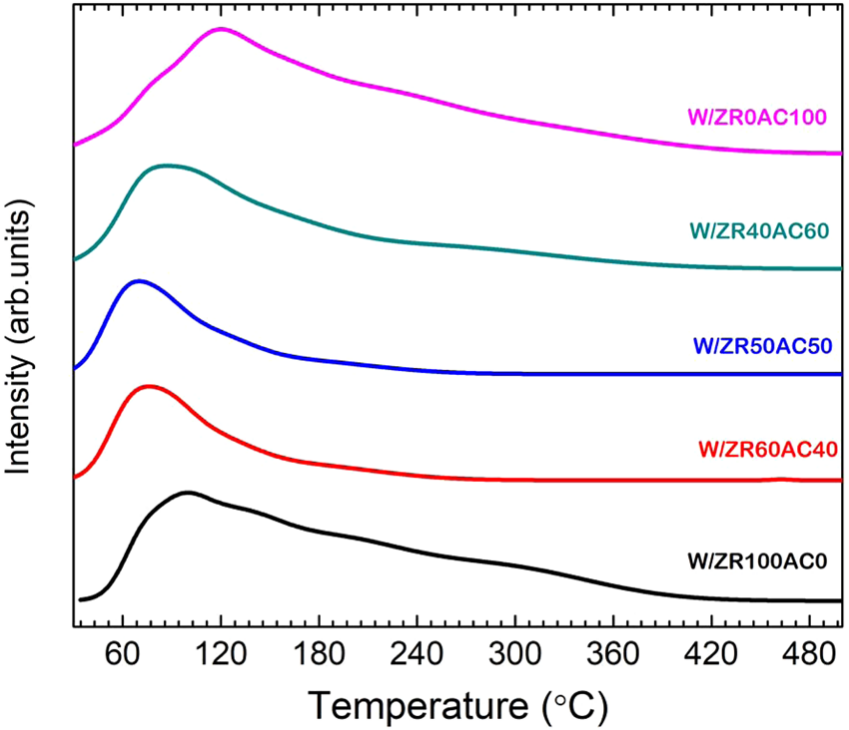
NH3-TPD technique of WO_*x*_/ZrO_2_–AC catalysts.

The characterizations of the bi-support systems shown in Table 1 revealed that the amount of tetragonal phase (I_970_/I_805_) of ZrO_2_ in the bi-support system is lower than that of the single-support systems, where the presence of the tetragonal phase enhances the reactivity of the catalysts^19^. Thus, the bi-support system should exhibit low ethanol conversion. However, it can be observed from Fig. 3 that the conversion of most of the tungsten catalyst bi-support is higher than using AC or ZrO_2_ alone as support. Thus, the trend in acidity and surface area is analyzed via the measurement from the temperature-programmed desorption of ammonia (NH_3_-TPD) in Fig. 4 if these may explain the high conversion found in the bi-support system. The total amounts of acid sites are estimated by the integration of NH_3_ desorption peaks. Almost all catalysts have large NH_3_ desorption profile area except for the 50:50 and 60:40 W/ZR:AC. The Acidity in μmol H^+^/g is illustrated in Table 1. The acidity of all bi-support systems is lower than both AC and ZrO_2_ single-support systems. Thus, this low acidity should reduce the activity of the catalyst, thus lower the conversion. However, the acidity trend is in contrast to the trend of ethanol conversion.

As a result, further insights on the characteristics of the catalyst on these bi-support surfaces are probed by SEM. It was revealed that the tungsten active sites are highly distributed on the zirconia single support system and all bi-support systems with high zirconia contents, while the pure activated carbon single support system and the bi-support with high activated content show poor distribution as can be seen in the EDX elemental mapping in Fig. 2(a2–e2). The good dispersion of active sites on zirconia containing systems may contribute to the high conversion found in the bi-support systems.

On the product selectivity in the ethanol dehydration reaction when (1) Ethylene, (2) Acetaldehyde and (3) Diethyl ether (DEE) are considered as dominant products, as shown in Fig. 3, it was found that the system with pure zirconia has high ethylene selectivity vary around 58 to 78%, while the rest of the product are acetaldehyde and diethyl ether. Despite the high ethylene selectivity found in pure zirconia single support system, the bi-support system with the addition of only 20 wt% activated carbon could significantly improve such selectivity up to almost 100%. Thus, when we use the zirconia as a support, we will obtain both ethylene and acetaldehyde as the main products, while using activated carbon as the support, only ethylene is the main product. It is hypothesized that the added carbon atoms may play a role in modifications of the catalyst surface via the electronic effects induced by carbon atoms on the tungsten active sites. Therefore the hypothesis is verified in the following section employing the density functional theory (DFT)-based analysis.

### Electronic properties

**Figure 5:**
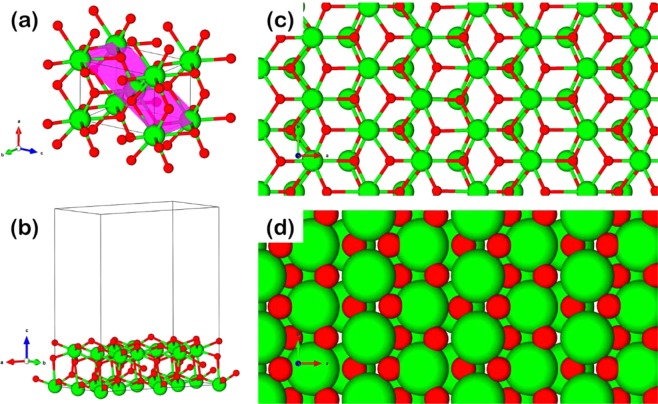
The ZrO_2_ presented as tetragonal-bulk structure with (**a**) (1 0 1) highlighted surface, (**b**) (1 0 1) slab model with vacuum 10 Å, top view of (1 0 1) surface with ball-stick (**c**) and space-filling view (**d**). Green and red atoms are Zr and O, respectively.

In this section, we used the DFT approach to investigate the role of activated carbon in the bi-support system of the tungsten catalyst, where high ethylene selectivity is observed. To illustrate the catalyst, the tungsten catalysts supported on the single ZrO_2_, and on the bi-support of ZrO_2_-activated-carbon were modeled via the WO_*x*_/ZrO_2_ and WO_*x*_/ZrO_2_–AC slab models based on ZrO_2_ crystal structure shown in Fig. 5(a). For the optimized ZrO_2_ slab model, the two-layer ZrO_2_(1 0 1) is chosen as this is suggested to be the most stable facet based on Tosoni *et al*.^20^. The ZrO_2_(1 0 1) surface is illustrated by the ball-and-stick, and space-filling models projected as the top view is shown in Fig. 5(c,d), respectively. The Fig. 5(b) shows the slab model of ZrO_2_(1 0 1) with the dimension cell 12.8874 Å × 7.2876 Å × 16.000 Å with the vacuum of 10 Å in *c*-direction taken to avoid the confinement effects from the interaction between the adsorbed ethylene molecule and the top periodic cell itself. Afterward, we constructed the WO_*x*_/ZrO_2_ model from the following procedure; (1) place the W atom onto the Zr and/or O atom and optimize to determine the most stable configuration, (2) place the O atoms, atom-by-atom around the W atom and optimize to generate the WO_*x*_ active site on the ZrO_2_ support, (3) place the ethylene molecule that is separately optimized in the vacuum to adsorb on the O atoms of the WO_*x*_ active site and optimize, and (4) calculate the ethylene adsorption energy (${{\rm{E}}}_{{\rm{ads}}}({{\rm{C}}}_{2}{{\rm{H}}}_{4})$) via the following equation.

1 $${{\rm{E}}}_{{\rm{ads}}}({{\rm{C}}}_{2}{{\rm{H}}}_{4})={{\rm{E}}}_{{\rm{slab}}+{\rm{mol}}}-{{\rm{E}}}_{{\rm{slab}}}-{{\rm{E}}}_{{\rm{mol}}}$$

E_slab+mol_ = the total energy of the ethylene adsorbed slab model (WO_*x*_/ZrO_2_–C and WO_*x*_/ZrO_2_–C_2_)

E_slab_ the total energy of the clean slab model (WO_*x*_/ZrO_2_–C, and WO_*x*_/ZrO_2_–C_2_)

E_mol_ = the total energy of the ethylene molecule.

It was found that a W atom on top of the O atom is the most stable configuration. Moreover, the WO_5_/ZrO_2_ is the most stable configuration for the tungsten active site due to the bond length between W and O (as shown in page 2 of the supplementary document) that agrees with the work from Hardcastle *et al*.^21^.

**Figure 6:**
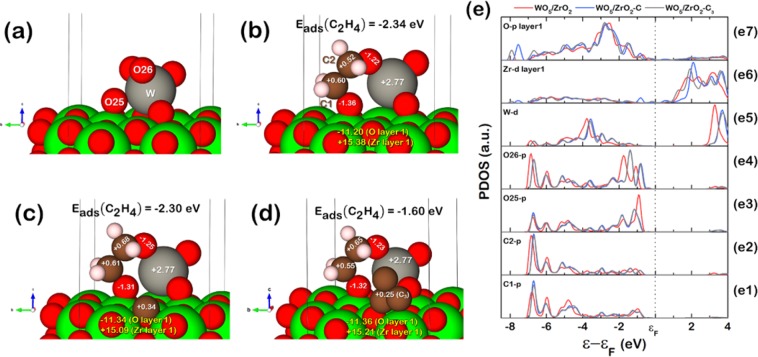
Charges transferred obtained from the Bader charge analysis in (**a**) WO_5_/ZrO_2_ (1 0 1) surface, (**b**) the Ethylene adsorption on WO_5_/ZrO_2_ and (**c**) ethylene adsorption on WO_5_/ZrO_2_–C, (**d**) ethylene adsorption on WO_5_/ZrO_2_–C_2_ and (**e**) projected density of states (PDOS) of (e1) p-orbital PDOS of C1 atom, (e2) p-orbital PDOS of C2 atom, (e3), p-orbital PDOS of O25 atom, (e4) p-orbital PDOS of O26 atom, (e5) d-orbital PDOS of W atom, (e6) d-orbital PDOS of the first layer Zr atoms, and (e7) p-orbital PDOS of the first layer O atoms.

The optimized (i) ethylene adsorbed WO_5_/ZrO_2_,(ii) ethylene adsorbed WO_5_/ZrO_2_, (iii) clean WO_5_/ZrO_2_–C, and (iv) clean WO_5_/ZrO_2_–C_3_ are shown in Fig. 6(a–d). The ethylene adsorption energy (${{\rm{E}}}_{{\rm{ads}}}({{\rm{C}}}_{2}{{\rm{H}}}_{4})$) is −2.34 eV for WO_5_/ZrO_2_ surface, −2.30 eV for WO_5_/ZrO_2_–C surface, and −1.60 eV for WO_5_/ZrO_2_–C_3_. Note that a highly negative value of ethylene adsorption energy corresponds to strong ethylene adsorption to the surface. Thus, the ethylene molecule is difficult to desorb from the surface. From the obtained adsorption energy for these three systems, the value decreases (weaker adsorption) when the carbon atom is added onto the WO_5_/ZrO_2_ surface at the ZrO_2_ region, indicating that the ethylene molecule on each bi-support system represented by both WO_5_/ZrO_2_–C and WO_5_/ZrO_2_–C_3_ surfaces is easier to desorb than that on the single support WO_5_/ZrO_2_ surface according to weaker adsorption energy. Therefore, the bi-support system comprises physically mixed zirconia and activated carbon exhibits high ethylene selectivity than that of the single ZrO_2_ support system due to weaker adsorption between ethylene and the surface. To get more insights into the situation around the WO_*x*_ adsorption site of ethylene, we carried out the Bader charge analysis^22–25^ and obtained the projected density of state (PDOS). We hypothesized that the carbon contributes to the electronic effects on either (i) adsorbed ethylene molecule, (ii) WO_5_ active site, or (iii) the WO_5_/ZrO_2_ surface as a whole. As a result, we analyze the charge transfers between the adsorbed ethylene molecule and the catalyst surface, as illustrated in Fig. 6(b–d) (also, the charge transfer for all atoms are shown in page 4-6 in the supplementary document). It revealed that the C1 and C2 atoms of ethylene lose more electron, in other words, becoming more positive after the carbon atom is introduced onto the surface around the ZrO_2_ region which agrees with the decreased ${{\rm{E}}}_{{\rm{ads}}}({{\rm{C}}}_{2}{{\rm{H}}}_{4})$, the less negative value of the adsorption energy found in the bi-support system models: WO_5_/ZrO_2_–C and WO_5_/ZrO_2_–C_3_.

The PDOS, considering the Fig. 6(e1–e7) corresponds to (i) C1 (in ethylene molecule), (ii) C2 (in ethylene molecule), (iii) O25 (surrounded the W atom), (iv) O26 (surrounded the W atom), (v) W, (vi) all the Zr atoms on the first layer of ZrO_2_(101) facet connecting to the O atoms, and (vii) all the O atoms on the first layer of ZrO_2_(101) connecting to the Zr atoms, accordingly. These PDOS profiles were plotted using their valence band, e.g., the oxygen atom has the valence band in the p orbital. Thus, the PDOS of p orbital is plotted for this element. The Fermi energy (*ε*_F_) was set to be at zero. Thus, the bonding and anti-bonding are located below and above *ε*_F_, respectively. In the C–C bonding, Fig. 6(e1,e2), shows that the PDOS of C1 and C2 shift up when compared to the single ZrO_2_ single-support system (red line) due to the increase in DOS peak at *ε* − *ε*_F_ = −7.0 eV after the carbon atoms are introduced onto the ZrO_2_ support (Blue and Grey lines). Such an upshift of peaks correlates with the charge transfers of both C1 and C2 carbon atoms, in which their charges decrease due to electron depletion. The upshift of the C1-p and C2-p PDOS is due to that their bonding with the active site becomes weaker. Thus, fewer electrons are in the bonding states, and more are in the antibonding states. Concerning the PDOS of the atoms, which are parts of the active site, the oxygen atoms connecting to the C1 and C2 atoms are considered. From the PDOS of O25, it shifted down and showed the intensity at around *ε* − *ε*_F_ = −1.0 to −1.5 eV. The downshift is also due to the added carbon atom. Despite the downshift, the bonding of O25 to the surface is still weak due to the shifting of PDOS on the surface, as shown in Fig. 6(d6,d7). It explains why the charge transfer of O25 decreases when the carbon atom is added onto the ZrO_2_ surface. The Zr-d PDOS profile shows a significant peak at *ε* − *ε*_F_ = −0.8 eV. Also, the PDOS of W-d, Zr-d, and O-p are shifted up. Although the charge transfer of O25 and O atoms around a W atom slightly decreased, the charge of the W atom is still constant. Figure 6(e7) corresponds to the first layer O atoms in the ZrO_2_ surface shows the peak at *ε* − *ε*_F_ = −7.8 eV for WO_5_/ZrO_2_–C and *ε* − *ε*_F_ = −8.0 eV for WO_5_/ZrO_2_–C_2_. Therefore, the charges transfer from the adsorbed ethylene molecule, O25, O26, and O atoms around W to the surface O atoms of the ZrO_2_ region. Hence, it is proposed that the carbon atom added onto the ZrO_2_ surface contributes to the induction of electron from the adsorbed ethylene to the catalyst surface, especially in the ZrO_2_ region, where this weakens the bond between the ethylene molecule and the tungsten active site in the ZrO_2_-activated carbon bi-support system.

Therefore, referring to the increase in the ethylene yield found in the bi-support system that comprises up to 50 wt% of AC is proposed to be due to the electronic effects of carbon atoms from AC on the catalyst. In addition, a high amount of AC would lead to a decrease in ethylene yield due to the deactivation via coking which is the common deactivation scheme found in reactions with carbonaceous reactants at high temperature^26^. Such deactivation causes the blockage of active sites, lowering the number of active sites resulting in a decrease in the total conversion of a catalyst.

## Conclusion

In this work, the high ethylene selectivity found on the zirconia-activated-carbon bi-support catalyst was investigated by both experimental and theoretical approaches. In the experiment, the activated carbon significantly enhances the ethylene selectivity when compared to the WO_*x*_/ZrO_2_ single-support system. The WO_*x*_/ZrO_2_50–AC50 shows the highest activity among the bi-support systems. The DFT investigation exposed that the WO_5_/ZrO_2_ single-support catalyst is suitable to represent to the tungsten on catalysts due to the agreement of bond length between W and O atom. The effects of the carbon in the bi-support system were further investigated via the Bader charge analysis of the charges induced on the ethylene molecule and the charges elsewhere on the active site of the catalyst together with the calculation of the projected density of state (PDOS). It was revealed that when the carbon atoms are introduced onto the zirconia support region, the ethylene adsorption energy is decreased due to the electron depletion in the carbon atoms of the ethylene molecule. The PDOS shows that the electron of ethylene’s carbon atoms that connect to the catalyst becomes weaker due to the induction of the electron transfer from the ethylene to the catalyst’s surface, specifically, the ZrO_2_ region. Therefore, the suggestions towards reactive and highly selective tungsten catalyst are that (1) the zirconia support should still be used to enhance the activity as it exhibits high ethanol conversion when compared with the system of activated carbon single support which only yields high conversion at significantly high temperature, (2) the activated carbon should be added to the pure zirconia supported tungsten catalyst to promote the product selectivity towards ethylene, while the system could be carried out at low enough temperature.

## Methodology

### Catalyst preparation and characterization

#### Preparation of support and bi-support

The zirconia support was prepared employing a precipitation technique as illustrated by Khaodee *et al*.^12^, in which the 0.15 M zirconyl nitrate [ZrO(NO_3_)_2_] was gradually added into the 2.5 wt% ammonium hydroxide (NH_4_OH) solution, while being stirred at 30 °C, where the pH of the solution is controlled at 10 throughout the preparation. The obtained precipitate was washed with deionized water until the chloride anion cannot be detected by the silver nitrate (AgNO_3_) solution. Afterward, this sample was dried overnight at 110 °C before the calcination at 450 °C for three h in air with a temperature ramp of 1 °C/min. For the activated carbon used for the activated carbon single support and added in the bi-support system, the commercial activated carbon was used.

The preparation of the bi-support began with the separated milling of zirconia and activated carbon before the physical mixing of both supports in a designated mass ratio followed by the fine milling. The activated carbon contents for the bi-support sample were varied as 20, 40, 50, 60 and 80 wt%, accordingly.

#### Catalyst preparation

The catalysts were prepared via the incipient wetness impregnation^11^. The ammonium metatungstate hydrate, (NH_4_)_6_H_2_W_12_O_40_·*x*H_2_O, was used as a precursor for the tungsten catalysts, where the percentage of tungsten in all support was designated at 15%. The obtained catalysts were dried overnight at 110 °C before being calcined under air at 500 °C for three h. The nomenclature assigned to each catalyst is WO_x_/ZrO_2_α–ACβ, where α and β are weight percentage of zirconia and activated carbon in the support, accordingly.

### Characterizations

#### Scanning electron microscopy (SEM) and energy-dispersive X-ray spectroscopy (EDX)

Morphologies of each catalyst were investigated by SEM, where JEOL model JSM-6400 was employed, while the elemental distribution of tungsten on the catalyst is determined via EDX.

#### Nitrogen adsorption-desorption isotherm

The surface area of each catalyst was calculated via the Brunauer-Emmett-Teller and BJH methods, where the 50 mg of sample was dried and outgassed in a cell at 200 °C at least 2 hours before being adsorbed by nitrogen. The adsorption was carried out employing liquid nitrogen at −196 °C in a Micromeritics Model Chemisorb 2750 automated system.

#### Acidity determination by NH_3_ temperature-programmed desorption

The flow apparatus was used to measure the acidity of each catalyst. Each sample of the catalyst of 0.1 g was treated in helium flowing at 50 cm^3^/min for one h, where the temperature was brought from room temperature of 30 °C to the calcination temperature of 500 °C at a rate of 20 °C/min. The acidity was calculated from the area of the profiles obtained from the Micromeritics Chemi Sorb 2750 pulse chemisorptions system analyzer. All peak fitting was carried out using Fityk program. The sum of all sub-peak areas was used to calculate the total amount of acid in each sample. To further confirm the trend in the acidity of all samples, ammonia leaving the apparatus was titrated with boric acid; then, the amount of acid in each sample was calculated.

#### Raman spectroscopy

The catalyst’s surface was investigated using the FT-Raman spectrometer, NXR FT-Raman model, from Thermo Scientific. The Raman spectra of the samples were collected at room temperature with the laser power of 50 mW, where the range scanned of 300–1200 cm^−1^ with a resolution of 16 cm^−1^ was employed.

### Reaction testing

The activity and selectivity of the catalysts were measured in the gas-phase ethanol dehydration reaction. From ethanol as a reactant, three main products are formed: acetaldehyde, ethylene, and DEE. The apparatus consists of a gas-phase ethanol generator, where ethanol was first evaporated and saturated in a three-necked flask before being carried by the argon carrier gas. The gas flow rate was controlled by the mass flow controller at 50 ml/min^27^. After this, saturated ethanol vapor was carried to a cylindrical glass tube reactor, where 0.05 g of catalyst was packed inside on 0.01 g quartz wool. The apparatus was operated employing the temperature-programmed technique, where the temperature of 200 °C was initially set as the starting temperature followed by the temperature of 250 °C, 300 °C, 350 °C, and 400 °C, accordingly. At each temperature, all products and left ethanol were detected and analyzed by Shimadzu GC-2014 gas chromatograph equipped with an FID detector, where the DB5 capillary column was used. The standard calibration curves for each component were used to calculate the amount of each component. The conversion and selectivity of each component were calculated via the following equations.

2 $${\rm{Conversion}}=\frac{{{\rm{Ethanol}}}_{{\rm{initial}}}\mbox{--}{{\rm{Ethanol}}}_{{\rm{final}}}}{{{\rm{Ethanol}}}_{{\rm{final}}}}\times 100 \% $$

3 $${{\rm{Selectivity}}}_{{\rm{Ethylene}}}=\frac{{{\rm{Ethylene}}}_{{\rm{out}}}}{{{\rm{Ethylene}}}_{{\rm{out}}}+{{\rm{Acetaldehyde}}}_{{\rm{out}}}+{{\rm{DEE}}}_{{\rm{out}}}}\times 100 \% $$

4 $${{\rm{Selectivity}}}_{{\rm{Acetaldehyde}}}=\frac{{{\rm{Acetaldehyde}}}_{{\rm{out}}}}{{{\rm{Ethylene}}}_{{\rm{out}}}+{{\rm{Acetaldehyde}}}_{{\rm{out}}}+{{\rm{DEE}}}_{{\rm{out}}}}\times 100 \% $$

5 $${{\rm{Selectivity}}}_{{\rm{DEE}}}=\frac{{{\rm{DEE}}}_{{\rm{out}}}}{{{\rm{Ethylene}}}_{{\rm{out}}}+{{\rm{Acetaldehyde}}}_{{\rm{out}}}+{{\rm{DEE}}}_{{\rm{out}}}}\times 100 \% $$

### Computational details

To expand why high ethylene selectivity was found in the zirconia-activated-carbon bi-support catalyst system, we performed the DFT using the projector augmented wave (PAW) implemented in the Vienna ab initio simulation package (VASP)^28–31^. The exchange-correlation function, along with the generalized gradient approximation (GGA) and Perdew, Burke, and Ernzerhof (PBE) function, especially the GGA+U was used^32^. In GGA+U function, the parameter U = 4.0 is used for Zr^20^ and W^33^, while the parameter J = 1.0 is used for W^33^. The cut-off energy of 350 eV and 3 × 3 × 1 Monkhorst-Pack *k*-mesh Brillouin-zone integration^34^ are used for all calculations. The structural optimization was performed within the conjugate gradient method^35^ and relaxed until the force converged less than 0.01 eV/Å, where structure figures were plotted using the VESTA package^36^.

## Data availability

The authors declare that relevant data are within the manuscript.

## Supplementary information

Supplementary Document
