# Supplementary material for: A computational-experimental investigation on high ethylene selectivity in ethanol dehydration reaction found on WOx/ZrO2-activated carbon bi-support systems
Source: Sci Rep. 2019 Dec 24;9:19738. doi: 10.1038/s41598-019-56373-3 (PMC6930307; doi:10.1038/s41598-019-56373-3)
Supplement: Supplementary file 1 — Supplementary Document [file 41598_2019_56373_MOESM1_ESM.pdf]

Supplementary document

A computational-experimental investigation on high ethylene selectivity in ethanol dehydration reaction found on WO_x/ZrO_2-activated carbon bi-support systems

Meena Rittiruam[1,2], Bunjerd Jongsomjit[2], and Supareak Praserthdam[1,2,*]

[1]*High-Performance Computing Unit (CECC-HCU), Center of Excellence in Catalysis and Catalytic Reaction Engineering (CECC), Chulalongkorn University, Bangkok, Thailand*

[2]*Center of Excellence in Catalysis and Catalytic Reaction Engineering (CECC), Chulalongkorn University, Bangkok, Thailand*

*Corresponding author's email: supareak.p@chula.ac.th (Dr. Supareak Praserthdam)

Table of content

Contents **Page**

Constructed WO_x/ZrO_2 model 2
Ethylene adsorption on WO_x/ZrO_2-C_y model 3
Bader charge and Charge transfer of C_2H_4 adsorption on WO_5/ZrO_2 4
Bader charge and Charge transfer of C_2H_4 adsorption on WO_5/ZrO_2-C 5
Bader charge and Charge transfer of C_2H_4 adsorption on WO_5/ZrO_2-C_3 6
POSCAR (for VASP and VESTA) WO2/ZrO2 and WO3/ZrO2 7
POSCAR (for VASP and VESTA) WO4/ZrO2 and C2H4 adsorption on WO4/ZrO2 8
POSCAR (for VASP and VESTA) C2H4 adsorption on WO5/ZrO2 9
POSCAR (for VASP and VESTA) C2H4 adsorption on WO5/ZrO2-C 10
POSCAR (for VASP and VESTA) C2H4 adsorption on WO5/ZrO2-C3 11
Figure S1. Ethylene yield of WO_x/ZrO_2–AC catalysts at various temperatures. 12

Constructed WO_x/ZrO_2 model

Model	Bond length (Å)
WO_2/ZrO_2 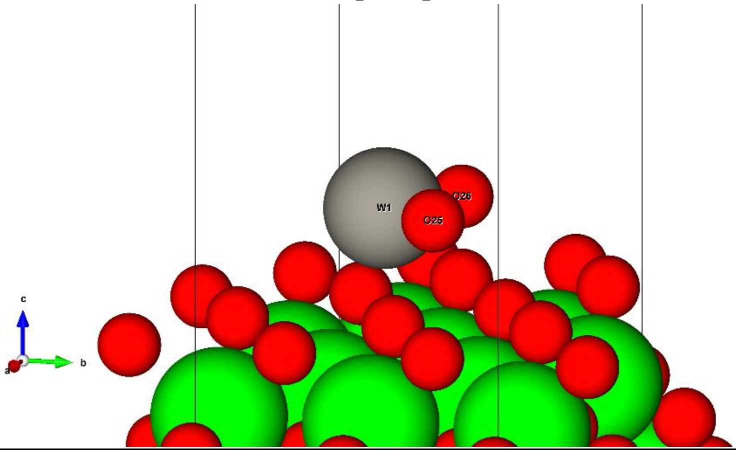	O25-W = 2.0517<br>O26-W = 2.0577<br><br>Reference: W-O = 1.7850 *
WO_3/ZrO_2 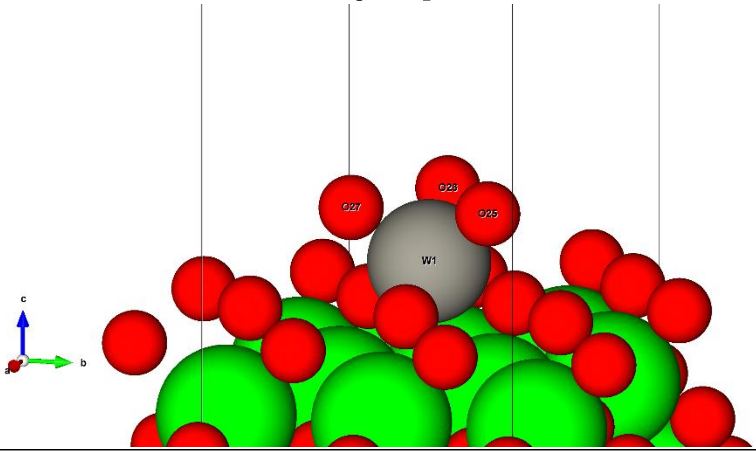	O25-W = 2.2415<br>O26-W = 2.1649<br>O27-W = 2.2388<br><br>Reference: W-O = 1.7850 *
WO_4/ZrO_2 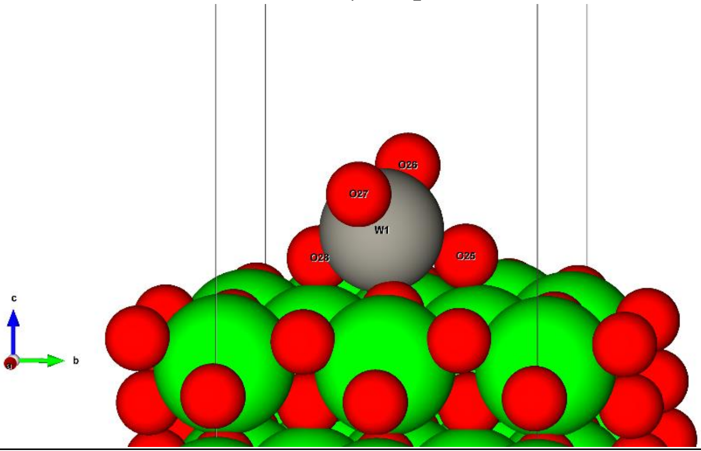	O25-W = 2.0636<br>O26-W = 1.9096<br>O27-W = 1.7323<br>O28-W = 1.8103<br><br>Reference: W-O = 1.7850 *

*F. Hardcastle, R. Lykins, Bond Length and Bond Valence for Tungsten-Oxygen and Tungsten-Sulfur Bonds, Journal of the Arkansas Academy of Science, 71 (2017) 59-61.

Ethylene adsorption on WO_x/ZrO_2-C_y model

Model	Ethylene adsorption energy (eV)
C_2H_4 adsorption on WO_4/ZrO_2 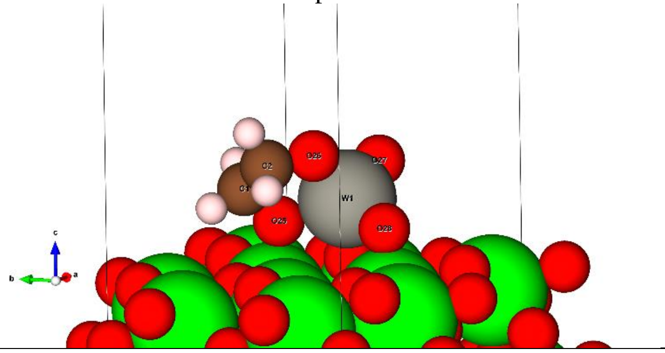	+0.16
C_2H_4 adsorption on WO_5/ZrO_2 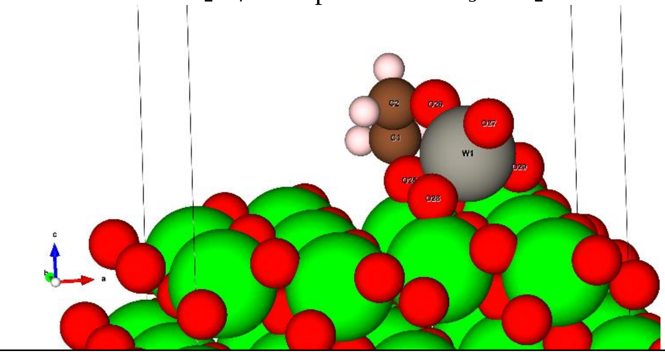	-2.34
C_2H_4 adsorption on WO_5/ZrO_2-C 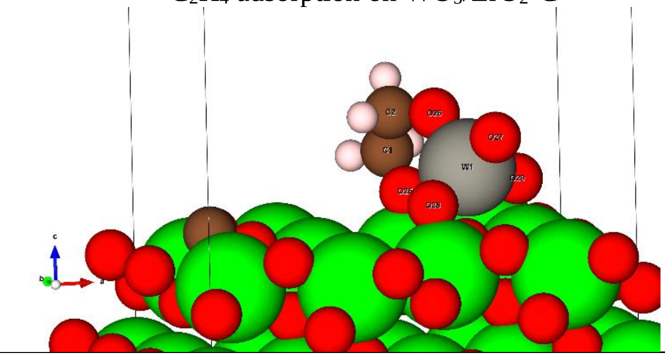	-2.30
C_2H_4 adsorption on WO_5/ZrO_2-C_3 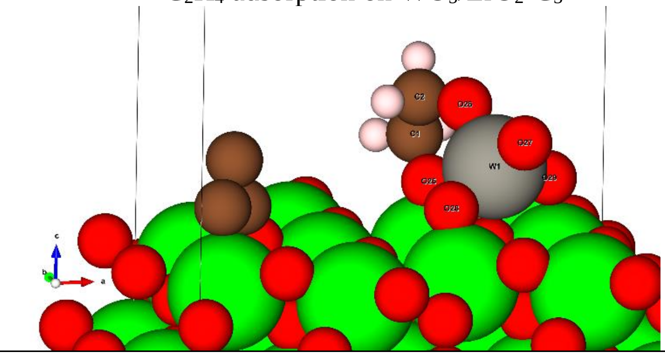	-1.60

Bader charge and Charge transfer of C_2H_4 adsorption on WO_5/ZrO_2

Label	No.	CHARGE	MIN DIST.	ATOMIC VOL.	Charge transfer
Zr1	1	9.465	0.9343	12.5692	2.535
Zr2	2	9.4158	0.9478	14.2475	2.5842
Zr3	3	10.2687	0.9382	63.7937	1.7313
Zr4	4	10.9555	1	78.3315	1.0445
Zr5	5	9.4715	0.9239	12.6213	2.5285
Zr6	6	9.3924	0.9478	10.3614	2.6076
Zr7	7	10.2242	0.9382	51.3713	1.7758
Zr8	8	10.8732	1	66.583	1.1268
Zr9	9	9.4577	0.9449	12.6377	2.5423
Zr10	10	9.4196	0.9478	14.5247	2.5804
Zr11	11	10.2584	0.9382	57.9456	1.7416
Zr12	12	10.8489	1	70.568	1.1511
Zr13	13	9.456	0.9015	10.6865	2.544
Zr14	14	9.3996	0.9478	10.3995	2.6004
Zr15	15	10.2199	0.9382	52.5964	1.7801
Zr16	16	10.7557	1	62.0154	1.2443
O1	17	7.3654	0.8528	16.5629	-1.3654
O2	18	7.3441	0.8499	14.6323	-1.3441
O3	19	7.2554	0.8743	12.1224	-1.2554
O4	20	7.2871	0.8587	13.5366	-1.2871
O5	21	7.24	0.8533	22.5925	-1.24
O6	22	7.2804	0.8723	54.7161	-1.2804
O7	23	7.3519	0.8528	16.1563	-1.3519
O8	24	7.3495	0.8499	15.0073	-1.3495
O9	25	7.2285	0.8743	11.8778	-1.2285
O10	26	7.2909	0.8605	13.0029	-1.2909
O11	27	7.2588	0.8378	24.2655	-1.2588
O12	28	7.2017	0.8186	16.9803	-1.2017
O13	29	7.3605	0.8528	16.3389	-1.3605
O14	30	7.3449	0.8499	14.5812	-1.3449
O15	31	7.2543	0.8743	12.1311	-1.2543
O16	32	7.2594	0.8605	12.9833	-1.2594
O17	33	7.2362	0.8342	20.3805	-1.2362
O18	34	7.2696	0.8562	46.1852	-1.2696
O19	35	7.3486	0.8528	15.9281	-1.3486
O20	36	7.3565	0.8499	15.1345	-1.3565
O21	37	7.2294	0.8743	11.9311	-1.2294
O22	38	7.2597	0.8605	12.5268	-1.2597
O23	39	7.2592	0.8258	28.2798	-1.2592
O24	40	7.2018	0.8039	11.2582	-1.2018
O25	41	7.3569	0.8832	14.204	-1.3569
O26	42	7.2184	0.7386	45.1079	-1.2184
O27	43	6.8734	0.6644	106.1689	-0.8734
O28	44	7.0927	0.7257	20.137	-1.0927
O29	45	7.0039	0.6993	58.9261	-1.0039
W1	46	11.2268	0.7687	13.0627	2.7732
C1	47	3.3953	0.0417	8.9407	0.6047
C2	48	3.4775	0.1127	13.5181	0.5225
H1	49	0.9266	0.2678	67.8146	0.0734
H2	50	0.9184	0.2408	74.4813	0.0816
H3	51	0.9387	0.2103	21.6708	0.0613
H4	52	0.8554	0.1957	8.295	0.1446

Bader charge and Charge transfer of C_2H_4 adsorption on WO_5/ZrO_2-C

Label	No.	CHARGE	MIN DIST.	ATOMIC VOL.	Charge transfer
Zr1	1	9.4669	0.9343	12.6061	2.5331
Zr2	2	9.5445	0.9478	12.492	2.4555
Zr3	3	10.2486	0.9382	62.8002	1.7514
Zr4	4	10.8257	1	72.7084	1.1743
Zr5	5	9.4742	0.9239	12.5398	2.5258
Zr6	6	9.4186	0.9478	10.2212	2.5814
Zr7	7	10.2263	0.9382	51.4974	1.7737
Zr8	8	10.7645	1	64.3122	1.2355
Zr9	9	9.4676	0.9449	12.8692	2.5324
Zr10	10	9.5412	0.9478	12.4181	2.4588
Zr11	11	10.2673	0.9382	57.3271	1.7327
Zr12	12	10.9481	1	73.3649	1.0519
Zr13	13	9.4511	0.9071	10.5191	2.5489
Zr14	14	9.3984	0.9478	10.4614	2.6016
Zr15	15	10.2271	0.9382	52.3627	1.7729
Zr16	16	10.8769	1	65.2971	1.1231
O1	17	7.3653	0.8528	16.3879	-1.3653
O2	18	7.349	0.8499	14.8976	-1.349
O3	19	7.272	0.8743	12.1637	-1.272
O4	20	7.2802	0.8587	13.5279	-1.2802
O5	21	7.2255	0.8518	23.6252	-1.2255
O6	22	7.2778	0.8723	46.4102	-1.2778
O7	23	7.3508	0.8528	16.2465	-1.3508
O8	24	7.3459	0.8499	14.8965	-1.3459
O9	25	7.2313	0.8743	11.9257	-1.2313
O10	26	7.2911	0.8605	13.0138	-1.2911
O11	27	7.2385	0.8378	21.3979	-1.2385
O12	28	7.206	0.8186	16.7042	-1.206
O13	29	7.3607	0.8528	16.3824	-1.3607
O14	30	7.3451	0.8499	14.8617	-1.3451
O15	31	7.2717	0.8743	12.155	-1.2717
O16	32	7.191	0.8605	11.5398	-1.191
O17	33	7.2364	0.8342	20.7381	-1.2364
O18	34	7.4393	0.8336	22.2349	-1.4393
O19	35	7.3522	0.8528	16.177	-1.3522
O20	36	7.3535	0.8499	14.9367	-1.3535
O21	37	7.2356	0.8743	12.0246	-1.2356
O22	38	7.266	0.8605	12.5018	-1.266
O23	39	7.2239	0.8258	23.4067	-1.2239
O24	40	7.2171	0.8222	10.6332	-1.2171
O25	41	7.3115	0.8719	13.5247	-1.3115
O26	42	7.2484	0.7457	58.0336	-1.2484
O27	43	6.8466	0.6539	88.6571	-0.8466
O28	44	7.0877	0.7429	20.2359	-1.0877
O29	45	6.9973	0.7132	48.1907	-0.9973
W1	46	11.2304	0.7711	11.9681	2.7696
C1	47	3.3855	0.1295	8.9374	0.6145
C2	48	3.3236	0.0739	12.3583	0.6764
C3	49	3.6633	0.0257	58.8011	0.3367
H	50	0.9177	0.2221	69.9136	0.0823
H	51	1.0324	0.3114	80.3718	-0.0324
H	52	1.0285	0.2933	20.6261	-0.0285
H	53	0.8543	0.2367	8.4874	0.1457

Bader charge and Charge transfer of C_2H_4 adsorption on WO_5/ZrO_2-C_3

Label	No.	CHARGE	MIN DIST.	ATOMIC VOL.	Charge transfer
Zr1	1	9.4703	0.9343	12.6061	2.5297
Zr2	2	9.5219	0.9478	10.8767	2.4781
Zr3	3	10.2542	0.9382	60.5707	1.7458
Zr4	4	10.8156	1	65.7406	1.1844
Zr5	5	9.4706	0.9239	12.3507	2.5294
Zr6	6	9.4213	0.9478	10.206	2.5787
Zr7	7	10.2245	0.9382	51.8866	1.7755
Zr8	8	10.7476	1	62.0697	1.2524
Zr9	9	9.4622	0.9449	12.6485	2.5378
Zr10	10	9.4428	0.9478	13.6649	2.5572
Zr11	11	10.2478	0.9382	55.2204	1.7522
Zr12	12	10.9498	1	69.4158	1.0502
Zr13	13	9.4546	0.9239	10.4647	2.5454
Zr14	14	9.3981	0.9478	10.4756	2.6019
Zr15	15	10.2272	0.9382	52.2279	1.7728
Zr16	16	10.8735	1	63.772	1.1265
O1	17	7.3655	0.8528	16.4596	4.6345
O2	18	7.3506	0.8499	14.953	4.6494
O3	19	7.2737	0.8743	12.1507	4.7263
O4	20	7.2646	0.8587	13.1812	4.7354
O5	21	7.2205	0.8518	21.1175	4.7795
O6	22	7.2449	0.8723	27.3102	4.7551
O7	23	7.3534	0.8528	16.3922	4.6466
O8	24	7.345	0.8499	14.966	4.655
O9	25	7.23	0.8743	11.8974	4.77
O10	26	7.2906	0.8605	13.0268	4.7094
O11	27	7.2193	0.8378	17.1607	4.7807
O12	28	7.2027	0.8186	16.4716	4.7973
O13	29	7.3603	0.8528	16.4716	4.6397
O14	30	7.3459	0.8499	14.9389	4.6541
O15	31	7.2664	0.8743	12.1572	4.7336
O16	32	7.2714	0.8605	12.6855	4.7286
O17	33	7.2344	0.8342	20.7838	4.7656
O18	34	7.5212	0.8385	14.7497	4.4788
O19	35	7.3547	0.8528	16.2965	4.6453
O20	36	7.3567	0.8499	15.0519	4.6433
O21	37	7.2387	0.8743	12.0463	4.7613
O22	38	7.2656	0.8605	12.5039	4.7344
O23	39	7.2379	0.8258	24.2622	-1.2379
O24	40	7.2151	0.8255	10.5191	-1.2151
O25	41	7.3201	0.8762	13.6518	-1.3201
O26	42	7.2345	0.7425	57.6086	-1.2345
O27	43	6.8457	0.6504	67.3646	-0.8457
O28	44	7.0833	0.7478	20.6044	-1.0833
O29	45	7.0051	0.7024	29.918	-1.0051
W1	46	11.2246	0.7676	11.9409	2.7754
C1	47	3.4468	0.0633	9.3059	0.5532
C2	48	3.3494	0.0726	12.7018	0.6506
C3	49	6.3374	0.0769	27.1732	-2.3374
C4	50	3.1577	0.0359	26.6232	0.8423
C5	51	2.2495	0.0191	123.3829	1.7505
H1	52	0.8918	0.2283	74.5954	0.1082
H2	53	1.045	0.3259	49.0407	-0.045
H3	54	0.9143	0.2265	18.4934	0.0857
H4	55	0.8839	0.2347	8.5363	0.1161

POSCAR (for VASP and VESTA) WO2/ZrO2 and WO3/ZrO2

WO_2/ZrO_2	WO_3/ZrO_2
1.00000	1.0000000000000000
12.8873996734999992 0 0	12.8873996734999992 0 0
0 7.2876000404000001 0	0 7.2876000404000001 0
0 0 16.0000000000000000	0 0 16.0000000000000000
Zr O W	Zr O W
16 26 1	16 27 1
Direct	Direct
0.3339900020000002 0.0000000000000000 0.1909600050000009	0.3339900020000002 0.0000000000000000 0.1909600050000009
0.0919900040000030 0.2500000000000000 0.1896000059999992	0.0919900040000030 0.2500000000000000 0.1896000059999992
0.0000000000000000 0.0000000000000000 0.0000000000000000	0.0000000000000000 0.0000000000000000 0.0000000000000000
0.2500000000000000 0.2500000000000000 0.0000000000000000	0.2500000000000000 0.2500000000000000 0.0000000000000000
0.8339899650000007 0.0000000000000000 0.1909600050000009	0.8339899650000007 0.0000000000000000 0.1909600050000009
0.5919899760000007 0.2500000000000000 0.1896000059999992	0.5919899760000007 0.2500000000000000 0.1896000059999992
0.5000000000000000 0.0000000000000000 0.0000000000000000	0.5000000000000000 0.0000000000000000 0.0000000000000000
0.7500000369999995 0.2500000000000000 0.0000000000000000	0.7500000369999995 0.2500000000000000 0.0000000000000000
0.3339900020000002 0.5000000000000000 0.1909600050000009	0.3339900020000002 0.5000000000000000 0.1909600050000009
0.0919900040000030 0.7500000160000013 0.1896000059999992	0.0919900040000030 0.7500000160000013 0.1896000059999992
0.0000000000000000 0.5000000000000000 0.0000000000000000	0.0000000000000000 0.5000000000000000 0.0000000000000000
0.2500000000000000 0.7500000160000013 0.0000000000000000	0.2500000000000000 0.7500000160000013 0.0000000000000000
0.8339899650000007 0.5000000000000000 0.1909600050000009	0.8339899650000007 0.5000000000000000 0.1909600050000009
0.5919899760000007 0.7500000160000013 0.1896000059999992	0.5919899760000007 0.7500000160000013 0.1896000059999992
0.5000000000000000 0.5000000000000000 0.0000000000000000	0.5000000000000000 0.5000000000000000 0.0000000000000000
0.7500000369999995 0.7500000160000013 0.0000000000000000	0.7500000369999995 0.7500000160000013 0.0000000000000000
0.0639000019999969 0.2500000000000000 0.0352899989999997	0.0639000019999969 0.2500000000000000 0.0352899989999997
0.3561600029999994 0.0000000000000000 0.0586300009999974	0.3561600029999994 0.0000000000000000 0.0586300009999974
0.2339500080000008 0.2500000000000000 0.1291999970000006	0.2339500080000008 0.2500000000000000 0.1291999970000006
0.0120400010000026 0.9925700140000018 0.1584900020000006	0.0120400010000026 0.9925700140000018 0.1584900020000006
0.4233525684425433 0.2730998603932530 0.3206251132679261	0.4249108530663988 0.2651830956906240 0.3110454293105508
0.1892573621457254 0.9775692061432194 0.3119326146835808	0.1838269366805249 0.0021443233299971 0.3082819265388448
0.5639000070000009 0.2500000000000000 0.0352899989999997	0.5639000070000009 0.2500000000000000 0.0352899989999997
0.8561600029999994 0.0000000000000000 0.0586300009999974	0.8561600029999994 0.0000000000000000 0.0586300009999974
0.7339499899999993 0.2500000000000000 0.1291999970000006	0.7339499899999993 0.2500000000000000 0.1291999970000006
0.5262299660000025 0.0000000000000000 0.1525399980000017	0.5262299660000025 0.0000000000000000 0.1525399980000017
0.9180780090794212 0.2551279034810522 0.3000845275269683	0.9172350140139053 0.2553116199492502 0.3003978642214390
0.6764386340968306 0.9860023960476604 0.3126743512123883	0.6723515001444650 0.0003604978532197 0.3223506892368743
0.0639000019999969 0.7500000160000013 0.0352899989999997	0.0639000019999969 0.7500000160000013 0.0352899989999997
0.3561600029999994 0.5000000000000000 0.0586300009999974	0.3561600029999994 0.5000000000000000 0.0586300009999974
0.2339500080000008 0.7500000160000013 0.1291999970000006	0.2339500080000008 0.7500000160000013 0.1291999970000006
0.0262300010000018 0.5000000000000000 0.1525399980000017	0.0262300010000018 0.5000000000000000 0.1525399980000017
0.4203911488231020 0.7481570776236351 0.3046316977955482	0.4250555134246369 0.7346991634042771 0.3109712628670805
0.1811825676400523 0.4890796261457955 0.3109672774629816	0.1903337977096039 0.4983780364001689 0.3139038283437876
0.5639000070000009 0.7500000160000013 0.0352899989999997	0.5639000070000009 0.7500000160000013 0.0352899989999997
0.8561600029999994 0.5000000000000000 0.0586300009999974	0.8561600029999994 0.5000000000000000 0.0586300009999974
0.7339499899999993 0.7500000160000013 0.1291999970000006	0.7339499899999993 0.7500000160000013 0.1291999970000006
0.5262299660000025 0.5000000000000000 0.1525399980000017	0.5262299660000025 0.5000000000000000 0.1525399980000017
0.9169095450283410 0.7427211794494147 0.3013550721259738	0.9157261745051725 0.7436659280472467 0.3007616335265690
0.6781498754565171 0.4997714901567226 0.3113132464800628	0.6683741918821966 0.5001541180189668 0.3197655247551785
0.5342403794181596 0.5634133101541590 0.4484441129445152	0.5777447177575519 0.7205440273868954 0.4597378460242902
0.3198778898745958 0.5567661186349683 0.4547596137209047	0.3834176342565609 0.5004434604955729 0.4659872738330497
0.4293264576319837 0.3516479167553415 0.4483606544319433	0.5774473821348521 0.2807767853933091 0.4599109706633404
	0.5110183488263038 0.5003890877342345 0.3779845189212060

POSCAR (for VASP and VESTA) WO4/ZrO2 and C2H4 adsorption on WO4/ZrO2

WO_4/ZrO_2

```
   1.0000000000000000
    12.8873996734999992    0   0
     0    7.2876000404000001    0
     0    0   16.0000000000000000
   Zr   O    W
    16    28     1
Direct
  0.3339900020000002  0.0000000000000000  0.1909600050000009
  0.0919900040000030  0.2500000000000000  0.1896000059999992
  0.0000000000000000  0.0000000000000000  0.0000000000000000
  0.2500000000000000  0.2500000000000000  0.0000000000000000
  0.8339899650000007  0.0000000000000000  0.1909600050000009
  0.5919899760000007  0.2500000000000000  0.1896000059999992
  0.5000000000000000  0.0000000000000000  0.0000000000000000
  0.7500000369999995  0.2500000000000000  0.0000000000000000
  0.3339900020000002  0.5000000000000000  0.1909600050000009
  0.0919900040000030  0.7500000160000013  0.1896000059999992
  0.0000000000000000  0.5000000000000000  0.0000000000000000
  0.2500000000000000  0.7500000160000013  0.0000000000000000
  0.8339899650000007  0.5000000000000000  0.1909600050000009
  0.5919899760000007  0.7500000160000013  0.1896000059999992
  0.5000000000000000  0.5000000000000000  0.0000000000000000
  0.7500000369999995  0.7500000160000013  0.0000000000000000
  0.0639000019999969  0.2500000000000000  0.0352899989999997
  0.3561600029999994  0.0000000000000000  0.0586300009999974
  0.2339500080000008  0.2500000000000000  0.1291999970000006
  0.0120400010000026  0.9925700140000018  0.1584900020000006
  0.4001471999067832  0.2488783277791313  0.2266822109675672
  0.1862277942846191  0.0003655065639023  0.2432706440822585
  0.5639000070000009  0.2500000000000000  0.0352899989999997
  0.8561600029999994  0.0000000000000000  0.0586300009999974
  0.7339499899999993  0.2500000000000000  0.1291999970000006
  0.5262299660000025  0.0000000000000000  0.1525399980000017
  0.9040032837418579  0.2565856980077470  0.2284351130929281
  0.6931782603934948  0.0063694729098671  0.2414351019471894
  0.0639000019999969  0.7500000160000013  0.0352899989999997
  0.3561600029999994  0.5000000000000000  0.0586300009999974
  0.2339500080000008  0.7500000160000013  0.1291999970000006
  0.0262300010000018  0.5000000000000000  0.1525399980000017
  0.3971036537973305  0.7507914736772463  0.2262113854840564
  0.1887835165406457  0.4957361840079386  0.2457094174069212
  0.5639000070000009  0.7500000160000013  0.0352899989999997
  0.8561600029999994  0.5000000000000000  0.0586300009999974
  0.7339499899999993  0.7500000160000013  0.1291999970000006
  0.5262299660000025  0.5000000000000000  0.1525399980000017
  0.8992376397368460  0.7408417853551228  0.2316518999269377
  0.6948455440714112  0.5012980843165572  0.2554334019245505
  0.5819865823752260  0.7121425584806863  0.3284946444621979
  0.5381828735919756  0.5251921522034703  0.4534480724249690
  0.7433843050126090  0.4043459264402974  0.4265924437045138
  0.5748277095114726  0.2562840519771510  0.3240203065597144
  0.6346863371375194  0.4592121308869537  0.3680056727091071
```

C_2H_4 adsorption on WO_4/ZrO_2

```
   1.0000000000000000
    12.8873996734999992    0   0
     0    7.2876000404000001    0
     0    0   16.0000000000000000
   Zr   O    W    C    H
    16    28     1     2     4
Direct
  0.3339900020000002  0.0000000000000000  0.1909600050000009
  0.0919900040000030  0.2500000000000000  0.1896000059999992
  0.0000000000000000  0.0000000000000000  0.0000000000000000
  0.2500000000000000  0.2500000000000000  0.0000000000000000
  0.8339899650000007  0.0000000000000000  0.1909600050000009
  0.5919899760000007  0.2500000000000000  0.1896000059999992
  0.5000000000000000  0.0000000000000000  0.0000000000000000
  0.7500000369999995  0.2500000000000000  0.0000000000000000
  0.3339900020000002  0.5000000000000000  0.1909600050000009
  0.0919900040000030  0.7500000160000013  0.1896000059999992
  0.0000000000000000  0.5000000000000000  0.0000000000000000
  0.2500000000000000  0.7500000160000013  0.0000000000000000
  0.8339899650000007  0.5000000000000000  0.1909600050000009
  0.5919899760000007  0.7500000160000013  0.1896000059999992
  0.5000000000000000  0.5000000000000000  0.0000000000000000
  0.7500000369999995  0.7500000160000013  0.0000000000000000
  0.0639000019999969  0.2500000000000000  0.0352899989999997
  0.3561600029999994  0.0000000000000000  0.0586300009999974
  0.2339500080000008  0.2500000000000000  0.1291999970000006
  0.0120400010000026  0.9925700140000018  0.1584900020000006
  0.4001471999067832  0.2488783277791313  0.2266822109675672
  0.1862277942846191  0.0003655065639023  0.2432706440822585
  0.5639000070000009  0.2500000000000000  0.0352899989999997
  0.8561600029999994  0.0000000000000000  0.0586300009999974
  0.7339499899999993  0.2500000000000000  0.1291999970000006
  0.5262299660000025  0.0000000000000000  0.1525399980000017
  0.9040032837418579  0.2565856980077470  0.2284351130929281
  0.6931782603934948  0.0063694729098671  0.2414351019471894
  0.0639000019999969  0.7500000160000013  0.0352899989999997
  0.3561600029999994  0.5000000000000000  0.0586300009999974
  0.2339500080000008  0.7500000160000013  0.1291999970000006
  0.0262300010000018  0.5000000000000000  0.1525399980000017
  0.3971036537973305  0.7507914736772463  0.2262113854840564
  0.1887835165406457  0.4957361840079386  0.2457094174069212
  0.5639000070000009  0.7500000160000013  0.0352899989999997
  0.8561600029999994  0.5000000000000000  0.0586300009999974
  0.7339499899999993  0.7500000160000013  0.1291999970000006
  0.5262299660000025  0.5000000000000000  0.1525399980000017
  0.8992376397368460  0.7408417853551228  0.2316518999269377
  0.6948455440714112  0.5012980843165572  0.2554334019245505
  0.5819865823752260  0.7121425584806863  0.3284946444621979
  0.5381828735919756  0.5251921522034703  0.4534480724249690
  0.7433843050126090  0.4043459264402974  0.4265924437045138
  0.5748277095114726  0.2562840519771510  0.3240203065597144
  0.6346863371375194  0.4592121308869537  0.3680056727091071
  0.5238016632912637  0.8140840790518180  0.3902554671773430
  0.4650204084552042  0.6700407664935892  0.4396978871915814
  0.4376082633608605  0.7218774690793693  0.5006068302229558
  0.3979091699904140  0.6172841705700252  0.4037876442684030
  0.4731119808888996  0.9139442380136725  0.3588689526803836
  0.5795821910631157  0.8884597042982111  0.4300794897528634
```

POSCAR (for VASP and VESTA) C2H4 adsorption on WO5/ZrO2

```
C2H4 adsorption on WO5/ZrO2
   1.0000000000000000
    12.8873996734999992    0    0
     0    7.2876000404000001    0
     0    0   16.0000000000000000
   Zr   O    W    C    H
    16    29     1     2     4
Selective dynamics
Direct
  0.3339900020000002  0.0000000000000000  0.1909600050000009
  0.0919900040000030  0.2500000000000000  0.1896000059999992
  0.0000000000000000  0.0000000000000000  0.0000000000000000
  0.2500000000000000  0.2500000000000000  0.0000000000000000
  0.8339899650000007  0.0000000000000000  0.1909600050000009
  0.5919899760000007  0.2500000000000000  0.1896000059999992
  0.5000000000000000  0.0000000000000000  0.0000000000000000
  0.7500000369999995  0.2500000000000000  0.0000000000000000
  0.3339900020000002  0.5000000000000000  0.1909600050000009
  0.0919900040000030  0.7500000160000013  0.1896000059999992
  0.0000000000000000  0.5000000000000000  0.0000000000000000
  0.2500000000000000  0.7500000160000013  0.0000000000000000
  0.8339899650000007  0.5000000000000000  0.1909600050000009
  0.5919899760000007  0.7500000160000013  0.1896000059999992
  0.5000000000000000  0.5000000000000000  0.0000000000000000
  0.7500000369999995  0.7500000160000013  0.0000000000000000
  0.0639000019999969  0.2500000000000000  0.0352899989999997
  0.3561600029999994  0.0000000000000000  0.0586300009999974
  0.2339500080000008  0.2500000000000000  0.1291999970000006
  0.0120400010000026  0.9925700140000018  0.1584900020000006
  0.4001471999067832  0.2488783277791313  0.2266822109675672
  0.1862277942846191  0.0003655065639023  0.2432706440822585
  0.5639000070000009  0.2500000000000000  0.0352899989999997
  0.8561600029999994  0.0000000000000000  0.0586300009999974
  0.7339499899999993  0.2500000000000000  0.1291999970000006
  0.5262299660000025  0.0000000000000000  0.1525399980000017
  0.9040032837418579  0.2565856980077470  0.2284351130929281
  0.6931782603934948  0.0063694729098671  0.2414351019471894
  0.0639000019999969  0.7500000160000013  0.0352899989999997
  0.3561600029999994  0.5000000000000000  0.0586300009999974
  0.2339500080000008  0.7500000160000013  0.1291999970000006
  0.0262300010000018  0.5000000000000000  0.1525399980000017
  0.3971036537973305  0.7507914736772463  0.2262113854840564
  0.1887835165406457  0.4957361840079386  0.2457094174069212
  0.5639000070000009  0.7500000160000013  0.0352899989999997
  0.8561600029999994  0.5000000000000000  0.0586300009999974
  0.7339499899999993  0.7500000160000013  0.1291999970000006
  0.5262299660000025  0.5000000000000000  0.1525399980000017
  0.8992376397368460  0.7408417853551228  0.2316518999269377
  0.6889691262238059  0.5003741582474730  0.2228750080801219
  0.5824923720289222  0.7198183734148573  0.3136266481061692
  0.6313786895698457  0.5771232081967975  0.4640604374560004
  0.7143126786970619  0.2310169828585049  0.4537835478239420
  0.5862356615765146  0.2883099734908183  0.3166169155772262
  0.8087274157342432  0.4736116455624131  0.3469712268472562
  0.6837871537800292  0.4064130957419032  0.3846936149394163
  0.5657886058981030  0.8336607723120307  0.3837518514555485
  0.5503180487107813  0.7100414189485208  0.4589512667990131
  0.5515783601058861  0.7914137070079330  0.5172690908781070
  0.4750111927656595  0.6372596691740083  0.4548022269743856
  0.4959436657146881  0.9213921940962300  0.3761162373830357
  0.6330556659709896  0.9254705071501959  0.3929532110810682
```

POSCAR (for VASP and VESTA) C2H4 adsorption on WO5/ZrO2-C

C_2H_4 adsorption on WO_5/ZrO_2-C

```
  1.0000000000000000
   12.8873996734999992    0   0
    0    7.2876000404000001    0
    0    0   16.0000000000000000
  Zr   O    W    C    H
   16    29    1    3    4
Selective dynamics
Direct
  0.3339900020000002  0.0000000000000000  0.1909600050000009
  0.0919900040000030  0.2500000000000000  0.1896000059999992
  0.0000000000000000  0.0000000000000000  0.0000000000000000
  0.2500000000000000  0.2500000000000000  0.0000000000000000
  0.8339899650000007  0.0000000000000000  0.1909600050000009
  0.5919899760000007  0.2500000000000000  0.1896000059999992
  0.5000000000000000  0.0000000000000000  0.0000000000000000
  0.7500000369999995  0.2500000000000000  0.0000000000000000
  0.3339900020000002  0.5000000000000000  0.1909600050000009
  0.0919900040000030  0.7500000160000013  0.1896000059999992
  0.0000000000000000  0.5000000000000000  0.0000000000000000
  0.2500000000000000  0.7500000160000013  0.0000000000000000
  0.8339899650000007  0.5000000000000000  0.1909600050000009
  0.5919899760000007  0.7500000160000013  0.1896000059999992
  0.5000000000000000  0.5000000000000000  0.0000000000000000
  0.7500000369999995  0.7500000160000013  0.0000000000000000
  0.0639000019999969  0.2500000000000000  0.0352899989999997
  0.3561600029999994  0.0000000000000000  0.0586300009999974
  0.2339500080000008  0.2500000000000000  0.1291999970000006
  0.0120400010000026  0.9925700140000018  0.1584900020000006
  0.4001471999067832  0.2488783277791313  0.2266822109675672
  0.1862277942846191  0.0003655065639023  0.2432706440822585
  0.5639000070000009  0.2500000000000000  0.0352899989999997
  0.8561600029999994  0.0000000000000000  0.0586300009999974
  0.7339499899999993  0.2500000000000000  0.1291999970000006
  0.5262299660000025  0.0000000000000000  0.1525399980000017
  0.9040032837418579  0.2565856980077470  0.2284351130929281
  0.6931782603934948  0.0063694729098671  0.2414351019471894
  0.0639000019999969  0.7500000160000013  0.0352899989999997
  0.3561600029999994  0.5000000000000000  0.0586300009999974
  0.2339500080000008  0.7500000160000013  0.1291999970000006
  0.0262300010000018  0.5000000000000000  0.1525399980000017
  0.3971036537973305  0.7507914736772463  0.2262113854840564
  0.1887835165406457  0.4957361840079386  0.2457094174069212
  0.5639000070000009  0.7500000160000013  0.0352899989999997
  0.8561600029999994  0.5000000000000000  0.0586300009999974
  0.7339499899999993  0.7500000160000013  0.1291999970000006
  0.5262299660000025  0.5000000000000000  0.1525399980000017
  0.8992376397368460  0.7408417853551228  0.2316518999269377
  0.6870910948340114  0.4943918226156016  0.2269264160057262
  0.5860412505747357  0.7136069645897896  0.3148258385747098
  0.6353128463920091  0.5792277174264103  0.4607616138668562
  0.7190366224911536  0.2496667706310648  0.4307893268797180
  0.5776326607756854  0.2977018565206469  0.3118346367611304
  0.8132169375320066  0.5075780372368122  0.3383048620579882
  0.6843654169372535  0.4346837744211915  0.3684557458695745
  0.5681606159220939  0.8354248102943889  0.3829439582608033
  0.5554227670696351  0.7143133349643257  0.4591481090037315
  0.0898002436593482  0.4987545391640846  0.2749528727266339
  0.5606367982255014  0.7962395389463022  0.5171396161812006
  0.4790255114750637  0.6443291341332102  0.4579213606739470
  0.4974128809282556  0.9191368404218725  0.3729473501068201
  0.6345641014043774  0.9301630893370048  0.3894655389653439
```

POSCAR (for VASP and VESTA) C2H4 adsorption on WO5/ZrO2-C3

```
C2H4 adsorption on WO5/ZrO2-C3
   1.0000000000000000
    12.8873996734999992    0    0
     0    7.2876000404000001    0
     0    0   16.0000000000000000
   Zr   O    W    C    H
    16    29     1     5     4
Selective dynamics
Direct
  0.3339900020000002  0.0000000000000000  0.1909600050000009
  0.0919900040000030  0.2500000000000000  0.1896000059999992
  0.0000000000000000  0.0000000000000000  0.0000000000000000
  0.2500000000000000  0.2500000000000000  0.0000000000000000
  0.8339899650000007  0.0000000000000000  0.1909600050000009
  0.5919899760000007  0.2500000000000000  0.1896000059999992
  0.5000000000000000  0.0000000000000000  0.0000000000000000
  0.7500000369999995  0.2500000000000000  0.0000000000000000
  0.3339900020000002  0.5000000000000000  0.1909600050000009
  0.0919900040000030  0.7500000160000013  0.1896000059999992
  0.0000000000000000  0.5000000000000000  0.0000000000000000
  0.2500000000000000  0.7500000160000013  0.0000000000000000
  0.8339899650000007  0.5000000000000000  0.1909600050000009
  0.5919899760000007  0.7500000160000013  0.1896000059999992
  0.5000000000000000  0.5000000000000000  0.0000000000000000
  0.7500000369999995  0.7500000160000013  0.0000000000000000
  0.0639000019999969  0.2500000000000000  0.0352899989999997
  0.3561600029999994  0.0000000000000000  0.0586300009999974
  0.2339500080000008  0.2500000000000000  0.1291999970000006
  0.0120400010000026  0.9925700140000018  0.1584900020000006
  0.4001471999067832  0.2488783277791313  0.2266822109675672
  0.1862277942846191  0.0003655065639023  0.2432706440822585
  0.5639000070000009  0.2500000000000000  0.0352899989999997
  0.8561600029999994  0.0000000000000000  0.0586300009999974
  0.7339499899999993  0.2500000000000000  0.1291999970000006
  0.5262299660000025  0.0000000000000000  0.1525399980000017
  0.9040032837418579  0.2565856980077470  0.2284351130929281
  0.6931782603934948  0.0063694729098671  0.2414351019471894
  0.0639000019999969  0.7500000160000013  0.0352899989999997
  0.3561600029999994  0.5000000000000000  0.0586300009999974
  0.2339500080000008  0.7500000160000013  0.1291999970000006
  0.0262300010000018  0.5000000000000000  0.1525399980000017
  0.3971036537973305  0.7507914736772463  0.2262113854840564
  0.1887835165406457  0.4957361840079386  0.2457094174069212
  0.5639000070000009  0.7500000160000013  0.0352899989999997
  0.8561600029999994  0.5000000000000000  0.0586300009999974
  0.7339499899999993  0.7500000160000013  0.1291999970000006
  0.5262299660000025  0.5000000000000000  0.1525399980000017
  0.8992376397368460  0.7408417853551228  0.2316518999269377
  0.6869003909874364  0.4926783248842029  0.2271032668065757
  0.5862286086430605  0.7142390101712693  0.3146523411741825
  0.6414521597679598  0.5757672071525988  0.4592650552261711
  0.7248255973688383  0.2456078126821277  0.4248240016993984
  0.5771467283321706  0.2985220322254425  0.3114412329695728
  0.8158920801645124  0.5105253161130613  0.3347862136190319
  0.6874370726402060  0.4335998139888121  0.3658163707970311
  0.5714493419760915  0.8343824946527139  0.3839393043181660
  0.5623386478989653  0.7126274123549879  0.4603484663680301
  0.1472806563008622  0.4171611993993736  0.3167691155737832
  0.0834759233822305  0.2686202016072031  0.3318548632632412
  0.1236025568947040  0.3896057324289226  0.3997815414606206
  0.5716210926602432  0.7944124969784305  0.5180321492648091
  0.4857338551398842  0.6436359774182143  0.4621755655756301
  0.5001441589378041  0.9178022674016777  0.3764546651135323
  0.6379575272849790  0.9293315054148289  0.3886694718032498
```

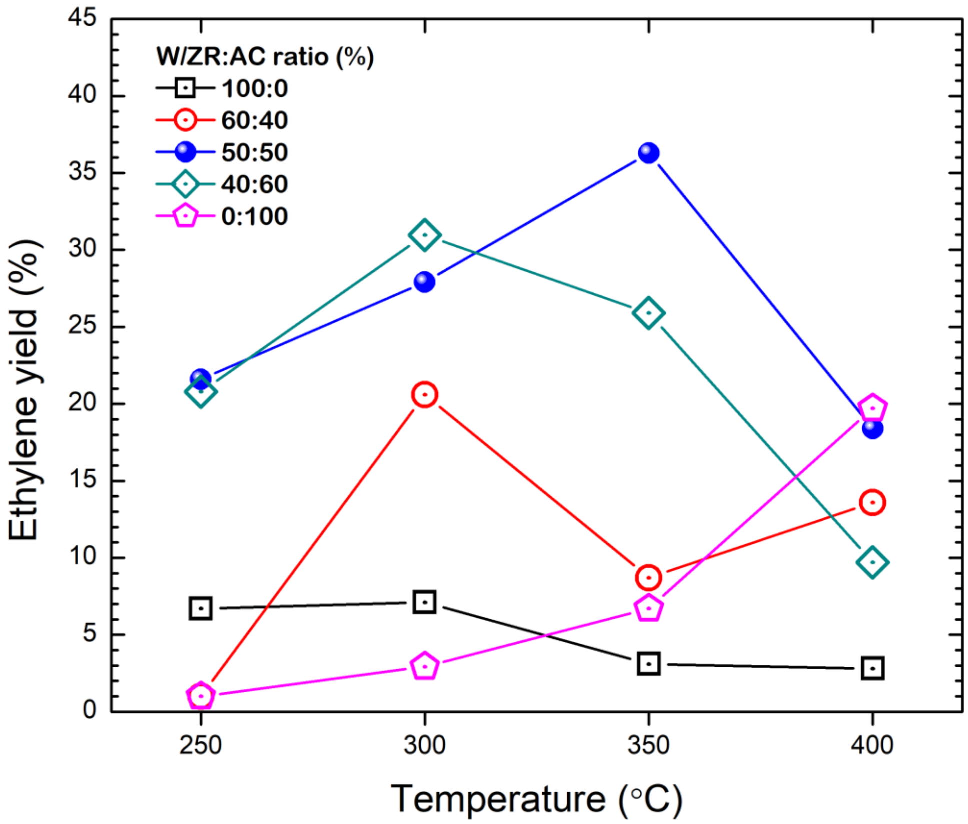

Figure S1. Ethylene yield of WO_x/ZrO_2–AC catalysts at various temperatures.
